# Case Report: Individualized circulating tumor DNA monitoring guides olaparib adjuvant therapy: an early-stage breast cancer case with somatic BRCA2 mutation

**DOI:** 10.3389/fonc.2026.1679086

**Published:** 2026-01-28

**Authors:** Qiuting You, Longlong Gong, Yi Chen, Xiaoxiao Dinglin, Ziliang Cheng, Qian Xia, Jinxia Xie, Jianli Zhao, Fengxi Su

**Affiliations:** 1 Breast Tumor Center, S’ Clinic, Guangzhou, China; 2 Genecast Biotechnology Co., Ltd, Wuxi, Jiangsu, China; 3 Breast Tumor Center, Sun Yat-Sen Memorial Hospital, Sun Yat-Sen University, Guangzhou, China; 4 Department of Radiology, Sun Yat-Sen Memorial Hospital, Sun Yat-Sen University, Guangzhou, China

**Keywords:** adjuvant therapy management, circulating tumor DNA, early-stage breast cancer, olaparib, somatic BRCA2 mutation

## Abstract

**Background:**

Circulating tumor DNA (ctDNA) has demonstrated a strong predictive capacity for recurrence in early-stage breast cancer compared with imaging examinations. However, there remains a paucity of robust clinical evidence to guide the adjustment of adjuvant therapy based on minimal residual disease (MRD) status in early-stage breast cancer.

**Case presentation:**

A 69-year-old female patient with early-stage triple-negative breast cancer (TNBC) with somatic BRCA2 mutations exhibited an exceptional response to adjuvant therapy with olaparib. Personalized ctDNA monitoring, utilizing a tumor-informed approach, was employed alongside imaging examinations and tumor biomarker testing to monitor tumor recurrence. MRD positivity was detected at four months and approximately one-month post-treatment discontinuation. Resumption of olaparib therapy resulted in a negative MRD status, while imaging examinations consistently demonstrated no evidence of recurrence in the patient.

**Conclusions:**

This report underscores the potential benefit of olaparib for early-stage TNBC patients with somatic BRCA2 mutations and the utility of serial ctDNA monitoring for tailoring individualized treatment strategies.

## Introduction

Breast cancer emerged as the most frequently diagnosed cancer worldwide in 2020, surpassing lung cancer for the first time in recorded history (1–3). In 2025, breast cancer diagnoses accounted for 32% of all cancer cases among women (4), and this proportion is projected to increase by a further 40% by 2040 (5). While significant improvements in breast cancer survival rates have been achieved through mammographic screening and adjuvant therapies (6, 7), breast cancer remains a leading cause of cancer-related mortality among women (1, 2). Adjuvant therapy plays a critical role in minimizing the risk of tumor recurrence following surgical resection. Currently, commercially available gene-based assays, such as Oncotype DX and MammaPrint, are utilized to predict the risk of recurrence in patients with hormone receptor (HR)+/human epidermal growth factor receptor 2 (HER2)- breast cancer and low tumor burden in axillary lymph nodes (8, 9). However, there remains no standardized approach to dynamically adjust adjuvant therapy in real-time, particularly at the time of resection, leading to potential undertreatment or overtreatment in some breast cancer patients. Recent clinical trials have demonstrated that minimal residual disease (MRD), as assessed by circulating tumor DNA (ctDNA), provides a sensitive and dynamic tool for monitoring disease recurrence in early-stage breast cancer (10–13). There is a critical need to better evaluate the impact of MRD on the management of adjuvant therapy.

This study investigates the role of MRD in predicting tumor recurrence and guiding adjuvant therapy in an early-stage triple-negative breast cancer (TNBC) patient harboring somatic BRCA2 mutations. MRD was assessed using a personalized panel targeting 50 somatic mutations identified via whole-exome sequencing (WES) of the primary tumor. This comprehensive approach aims to explore the potential of MRD-guided therapy in improving treatment outcomes for early-stage TNBC patients. Additionally, the efficacy of olaparib, a PARP inhibitor, in this TNBC case with somatic BRCA2 mutations during maintenance adjuvant therapy is discussed.

## Case report

**Figure 1:**
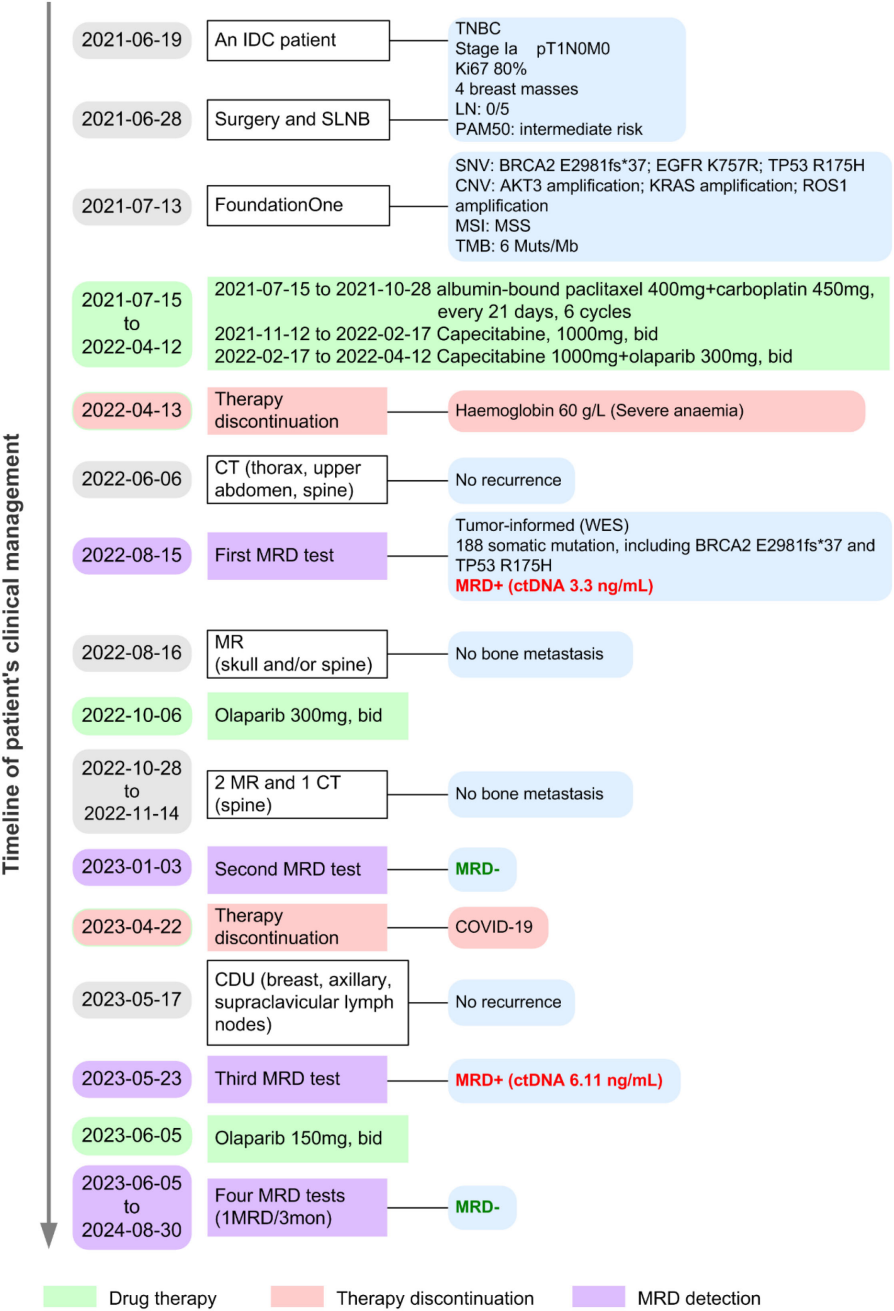
Clinical course of the patient with TNBC. CDU, color doppler ultrasound; CNV, copy number variant; ctDNA, circulating tumor DNA; CT, computed tomography; IDC, invasive ductal carcinoma; LN, lymph node; MRD, minimal residual disease; MR, magnetic resonance; MSS, microsatellite status stable; SLNB, sentinel lymph node biopsy; SNV, single nucleotide variant; WES, whole-exome sequencing.

**Figure 2:**
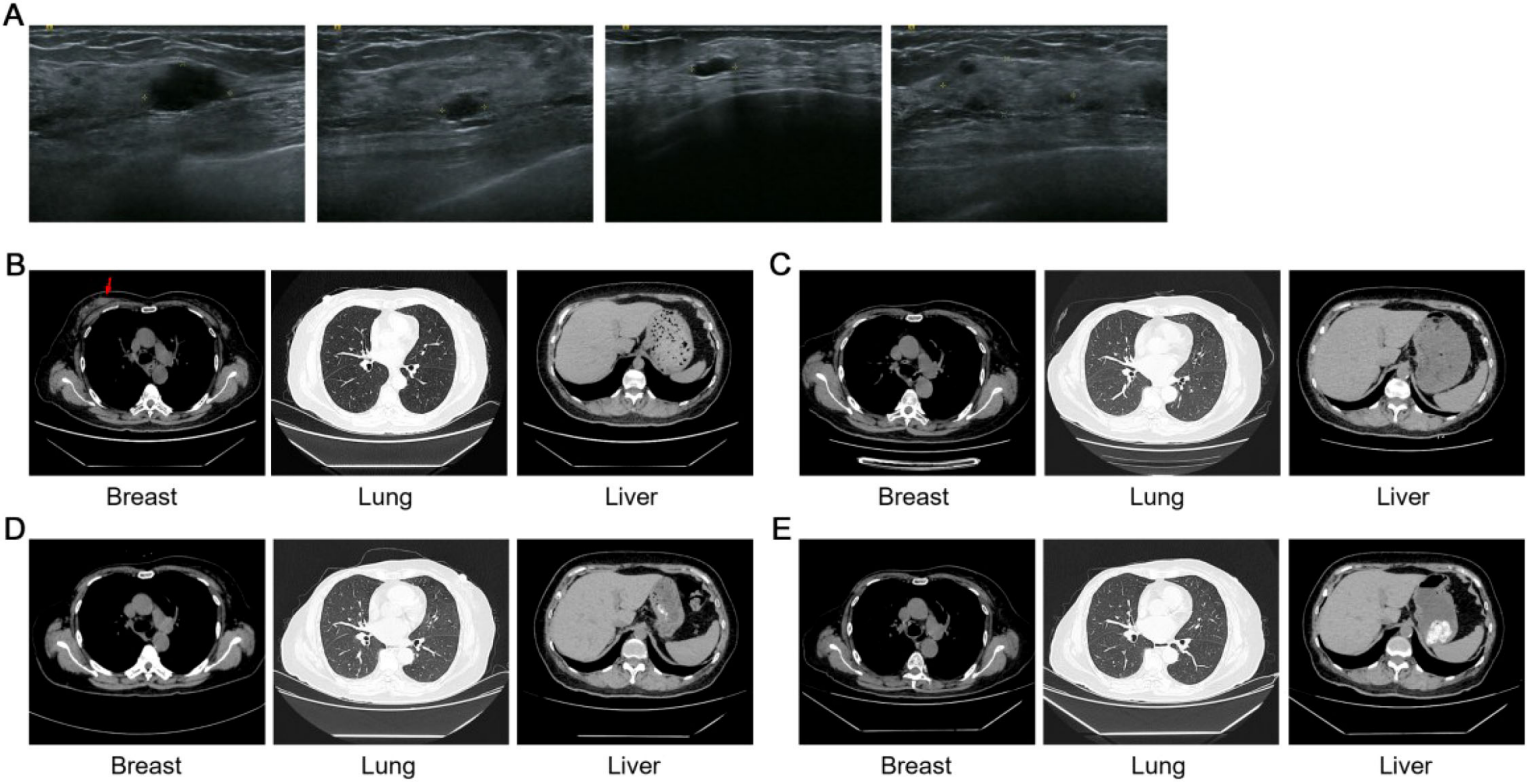
Imaging examination in the TNBC case. **(A)** Representative CDU images of the right breast. **(B-E)** Representative CT images of breast, lung and liver on June 22, 2021 **(B)**, June 6, 2022 **(C)**, July 20, 2023 **(D)** and July 19, 2024 **(E)**, respectively.

A 69-year-old postmenopausal female patient presented to the hospital on June 19, 2021, with a palpable mass in the right breast. Color doppler ultrasound (CDU) revealed multiple breast masses categorized as Breast Imaging Reporting and Data System (BI-RADS) 4C (**Figures 1**, **2A**). Mammography demonstrated clustered microcalcifications classified as BI-RADS 4B. No evidence of axillary lymph node enlargement or distant metastases was detected during physical and clinical examinations. A core needle biopsy (CNB) confirmed the diagnosis of triple-negative (TNBC), high-grade invasive ductal carcinoma (IDC, Nottingham grade 3). The clinical stage was T1N0M0 according to the American joint Committee on cancer (AJCC) tumor staging system. On June 28, 2021, the patient underwent a right mastectomy and sentinel lymph node biopsy (SLNB). Four breast tumors were excised, with sizes of 15×13×12 mm, 5×5×4 mm, 10×10×5 mm, and 8×8×5 mm, respectively. Histopathological examination confirmed the CNB findings, with no evidence of lymph node metastasis (LN 0/5). The pathological stage was T1N0M0 (AJCC stage Ia). PAM50 testing classified the tumor as basal-like breast cancer with an intermediate recurrence risk (PAM50 score: 55). FoundationOne CDx examination (Roche), a 324 gene panel detection of the tumor sample, identified three single nucleotide variants (SNVs; BRCA2 E2981fs*37, VAF 35.16%; EGFR K757R, VAF 56.52%; TP53 R175H, VAF 38.99%) and three copy number variants (CNVs; AKT3 amplification, CN 10; KRAS amplification, CN 7; ROS1 amplification, CN 8). The tumor exhibited a microsatellite-stable (MSS) phenotype with a tumor mutational burden (TMB) of 6 muts/Mb.

Adjuvant therapy was initiated following the standard anthracycline-cyclophosphamide and taxane (AC-T) in the patient with TNBC. Based on the SYSUCC-001 randomized clinical trial (14), capecitabine maintenance therapy for 1 year was performed in the early-stage TNBC. On the basis of the patient’s advanced age and concerns regarding anthracycline-related cardiac toxicity, a non-anthracycline-containing regimen was adopted. This decision was reached through detailed discussion with the patient. The patient successively received adjuvant therapy with 6 cycles of paclitaxel combined with carboplatin (TCb), and capecitabine maintenance therapy (**Figure 1**). Additionally, olaparib was administered as part of the adjuvant therapy regimen, despite being off-label for somatic BRCA2 mutations, with patient consent (**Figure 1**). This decision was informed by the findings of the OlympiA phase III trial (15), which demonstrated improved overall survival with olaparib in patients with germline BRCA1/2 mutations in early-stage high-risk breast cancer with specific pathological features (e.g., residual disease after neoadjuvant chemotherapy or a T2-T4 primary tumor at initial surgery received adjuvant chemotherapy). Given the established predictive value of BRCA2 inactivation for olaparib response and the association of MRD positivity with high recurrence risk, we proceeded with olaparib therapy after thorough discussion with the patient regarding potential benefits and limitations.

**Figure 3:**
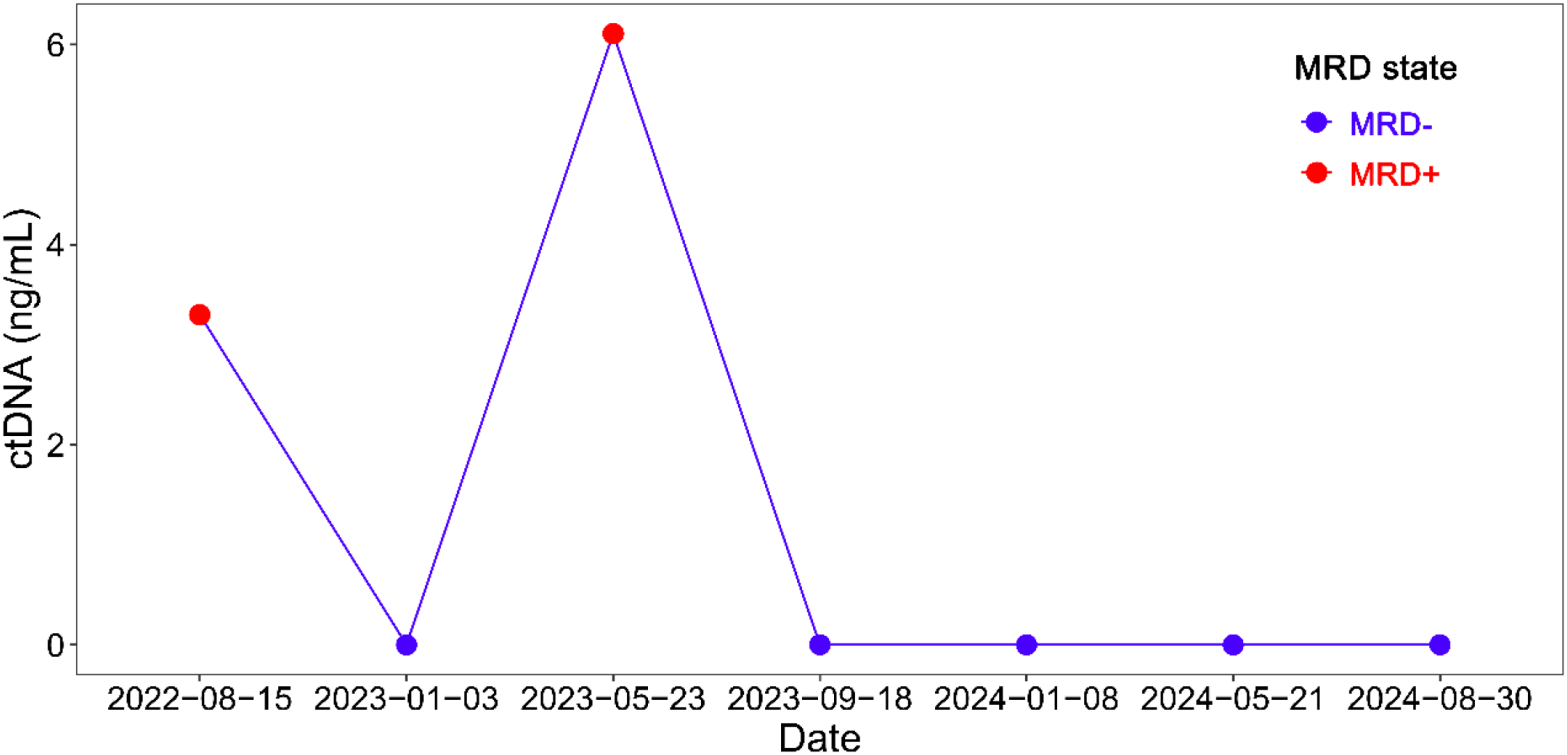
MRD dynamic monitoring for recurrence based on ctDNA in the case with TNBC.

During adjuvant therapy with capecitabine and olaparib, the patient developed severe anemia, necessitating treatment discontinuation on April 13, 2022. To monitor tumor recurrence and guide adjuvant therapy, serial MRD and imaging assessments were performed (**Figure 1**). A customized ctDNA panel targeting 50 genes with somatic mutations, identified via WES of the primary tumor, was employed for MRD monitoring. WES results identified 188 somatic mutations, including BRCA2 E2981fs*37 and TP53 R175H. The mean sequencing depth for MRD assays was >200,000×. The specific VAF values have now been provided across all time points in the **Supplementary Materials**. Four months after treatment discontinuation, MRD testing revealed positivity with ctDNA levels of 3.3 ng/mL (**Figure 3**), while thoracic, upper abdominal, and spinal computed tomography (CT) imaging indicated no evidence of recurrence (**Figures 2B, C**). Due to the severity of anemia, subsequent therapy consisted of olaparib monotherapy, which did not result in further adverse events. After three months of olaparib treatment, a second ctDNA assessment showed negative MRD results (**Figure 3**). However, treatment was again interrupted due to COVID-19 infection on April 22, 2023. Approximately one month after treatment discontinuation, CDU examinations of the breast, axillary, and supraclavicular lymph nodes revealed no signs of recurrence. CT imaging also showed no evidence of recurrence (**Figure 2D**). Concurrently, MRD testing was positive (ctDNA: 6.11 ng/mL), which was resolved to negative following 3.5 months of resumed olaparib therapy (**Figure 3**). Subsequent maintenance therapy with olaparib included quarterly MRD monitoring, with consistently negative results (**Figure 3**, **Supplementary Materials**), and imaging studies demonstrated no evidence of recurrence (**Figure 2E**).

To evaluate for potential brain and bone metastasis, whole-spine single photon emission computed tomography (SPECT) imaging was performed in June 2022, revealing mild degenerative changes in the right C4 transverse process and left C6 transverse process, costochondritis of the right 4th rib, osteoarthritis of the L4/L5 facet joints, and degenerative changes in the spine and right patella. Magnetic resonance imaging (MRI) and CT of the spine confirmed no evidence of bone metastasis, and MRI of the skull ruled out brain metastasis (data not shown).

## Discussion

TNBC, characterized by molecular heterogeneity and aggressive clinical behavior, remains a significant unmet medical need due to its poor prognosis and reliance on chemotherapy as the primary treatment modality (16). Current postoperative management strategies for TNBC are predominantly guided by conventional clinical staging and neoadjuvant treatment response, particularly the achievement of a pathological complete response (pCR), with a critical need for precision-oriented strategies to identify subpopulations most likely to benefit from escalated therapies. Recent advancements have demonstrated that MRD strongly predicts tumor recurrence in early-stage breast cancer. Studies have shown that MRD-positive patients exhibit a higher risk of recurrence and metastasis compared to MRD-negative patients (10–13). Raoul Charles Coombes’ research indicated that MRD can predict metastatic relapse up to 2 years in advance (median lead time of 8.9 months) in breast cancer patients across stages IA-IIIC (10). Heather A. Parsons’ studies revealed a median lead time of 18.9 months from the first positive MRD sample to recurrence in early-stage breast cancer (11) and 12.4 months in high-risk stage II-III HR+ breast cancer (12). MRD positivity was detected following treatment discontinuation, indicating a high risk of recurrence. However, traditional imaging modalities failed to detect abnormalities, underscoring the superior sensitivity of ctDNA in predicting tumor recurrence.

Emerging evidence suggests that MRD-directed treatment intensification may offer survival benefits. A recent study in colorectal cancer demonstrated the efficacy of adjuvant chemotherapy in MRD-positive patients with resected colorectal liver metastases (17). The ZEST trial evaluated whether poly (ADP-ribose) polymerase (PARP) inhibitors could enhance disease-free survival (DFS) in TNBC or HER2+ BRCA-mutated breast cancer patients with MRD (18). Although the trial was terminated early due to low ctDNA positivity rates, it indicated a trend toward improved DFS with PARP inhibitors in MRD-positive TNBC patients. In the present case, MRD status shifted from positive to negative following adjuvant therapy with olaparib. During subsequent long-term olaparib maintenance, MRD negativity was sustained, and no radiographic recurrence was detected. This case highlights the potential of MRD as a predictive biomarker for identifying patients who may benefit from intensified therapeutic regimens and targeted therapies.

PARP inhibitors, such as olaparib and talazoparib, have revolutionized treatment for breast cancer patients with germline BRCA1/2 mutations (15, 19–22) and recent trials suggest its efficacy extends to patients with somatic BRCA1/2 variants (23, 24). The TBCRC 048 trial demonstrated an objective response rate (ORR) of 50% in patients with somatic BRCA1/2 mutations treated with olaparib (23). In this TNBC patient with somatic BRCA2 mutations, MRD negativity and absence of radiographic recurrence were observed during olaparib adjuvant therapy. Despite MRD positivity following treatment discontinuations, resumption of olaparib led to sustained MRD negativity, with no evidence of recurrence as of the latest follow-up. This case also provides evidence supporting the use of olaparib in early-stage TNBC patients with somatic BRCA2 mutations.

While MRD detection offers significant advantages in predicting recurrence and guiding adjuvant therapy, several limitations must be considered. In clinical practice and exploratory studies, we observed that MRD examination might exhibit reduced sensitivity in breast cancer patients undergoing active adjuvant chemotherapy and cases of brain metastasis or local recurrence (25). In addition, the clonal hematopoiesis of indeterminate potential (CHIP) impacts on ctDNA accuracy (26). Despite these challenges, MRD technology holds substantial promise for enhancing breast cancer management. Numerous clinical trials, such as NCT03145961 and NCT04768426, have investigated MRD-guided adjuvant therapies in breast cancer and the results of these trials are still unreleased. Its clinical utility and reliability will be further validated through large-scale clinical trials.

## Conclusions

This report highlights the utility of MRD monitoring in guiding adjuvant therapy in an early-stage TNBC patient with somatic BRCA2 mutations and underscores the potential clinical benefit of olaparib in this population. Further validation through large-scale clinical trials is essential to confirm these observations.

## Data Availability

The datasets presented in this study can be found in online repositories. The names of the repository/repositories and accession number(s) can be found in the article/**Supplementary Material**.
